# Cyclopean, Dominant, and Non-dominant Gaze Tracking for Smooth Pursuit Gaze Interaction

**DOI:** 10.16910/jemr.10.1.2

**Published:** 2017-01-25

**Authors:** Tomer Elbaum, Michael Wagner, Assaf Botzer

**Affiliations:** Ariel University, Israel

**Keywords:** eye movement, gaze interaction, interactive eye tracking, smooth pursuit, usability, cyclopean eye, dominant eye, human-computer interaction

## Abstract

User-centered design questions in gaze interfaces have been explored in multitude empirical investigations. Interestingly, the question of what eye should be the input device has never been studied. We compared tracking accuracy between the “cyclopean” (i.e., midpoint between eyes) dominant and non-dominant eye. In two experiments, participants performed tracking tasks. In Experiment 1, participants did not use a crosshair. Results showed that mean distance from target was smaller with cyclopean than with dominant or non-dominant eyes. In Experiment 2, participants controlled a crosshair with their cyclopean, dominant and non-dominant eye intermittently and had to align the crosshair with the target. Overall tracking accuracy was highest with cyclopean eye, yet similar between cyclopean and dominant eye in the second half of the experiment. From a theoretical viewpoint, our findings correspond with the cyclopean eye theory of egocentric direction and provide indication for eye dominance, in accordance with the hemispheric laterality approach. From a practical viewpoint, we show that what eye to use as input should be a design consideration in gaze interfaces.

## Introduction

Eye-gaze interaction with computerized systems holds a number of benefits. For instance, users’ hands are free to perform other tasks while interacting with the computer ( 1 ) and individuals with severe motor disabilities can communicate with their environment more easily ( 2, 3 ). In addition, gaze interaction can be highly useful when screens are larger and when objects are in motion because time to move one's eyes between objects changes very little with distance ( 4 ) and tracking them when they are moving (as in video games) draws upon an inherent and especially adapt eye-brain mechanism ( 5, 6 ).

The interest in using gaze interfaces has led to empirical investigations of user-centered design questions. For instance, how should users select on-screen objects (e.g., icons) that they would like to interact with ( 7 )? Whether or not users should receive feedback on where they are looking ( 1 ) and what kind of feedback? ( 3, 8 ). Findings have shown that when users selected objects for interaction by dwelling on them for a certain duration, selection times were faster than with the "traditional" mouse ( 3, 4 ). Yet, other studies have demonstrated that when targets were smaller than 4° of visual angle, users had to confirm choices by key press or by moving their facial muscles to compete with the computer mouse in speed and in accuracy ( 9, 10 ). Finally, Alonso, Causse ( 1 ) found that for targets smaller than 2.14° , cursor feedback on where users were looking improved their accuracy in selecting these targets.

Interestingly, although pointing accuracy on smaller objects has been identified as key factor in the effectiveness of gaze interaction, the question of what eye points more accurately on targets has not been studied. This question may hold even greater importance in gaze interaction with moving targets that currently suffer from low success in target acquisition ( 10, 11 ). In the current study, we compared tracking accuracy between the “cyclopean”, dominant and non-dominant eye.

### Missing of targets and the higher accuracy of the “cyclopean eye”

Cui and Hondzinski ( 12 ) conducted an experiment where they tested the gaze accuracy of participants. Participants viewed targets (i.e., weighted fishing anchors) suspended from the ceiling at three different heights while their binocular points of gaze were recorded at 60Hz. Errors were quantified as the absolute and angular distances between targets and points of gaze of the right and of the left eye. Then, a third type of error was defined as the absolute and angular distances between targets and the average of the positions of the right and left eye. Findings showed that mean error of averaged positions were either smaller or not significantly different from the mean error of the right or of the left eye alone. Based on these findings, the conclusion from this study was that for a range of viewing conditions, averaged gaze positions would produce the most accurate results for viewing tasks.

From a broader theoretical perspective, Cui and Hondzinski ( 12 ) suggested that their findings resonate with the "cyclopean eye" theory that accounts for how people set their relative direction to objects in space. According to this theory, people set their egocentric visual direction according to a line connecting the target and a point on an imaginary line between their eyes. In other words, when one assesses her relative positions to targets, it is a point between her eyes that designates her position. This point was metaphorically termed the "cyclopean eye" ( 13 ) and numerous studies have indeed demonstrated that individuals set “cyclopean” direction to objects in their field of view (e.g., 14, 15, 16, 17 ). Cyclopean eye position, in turn, may be approximated by averaging left and right eye positions as in Cui and Hondzinski ( 12 ) study.

Although Cui and Hondzinski ( 12 ) did not account for why the right and left eye would miss targets in the first place, their findings do correspond with a well-documented phenomenon in optometry and the human vision and perception domains, termed “fixation disparity”. In fixation disparity, vergence eye movements fail to intersect both lines of sight on the intended targets and consequently, eyes do not land on the same spot, but rather fixate on slightly different locations from each other and from the intended targets ( 18, 19 ). Hence, while right and left eyes may sometimes miss targets, the "cyclopean eye", who sets the direction to targets, may be the one that is placed on them more accurately. Cyclopean eye theory, therefore, resonates with that averaged gaze positions, or cyclopean positions, may "land" closer to targets than single gaze positions. Still, another theory, that of eye dominance suggests that at least in some cases gaze positions of the dominant eye may land closer to targets.

### Eye dominance

The concept of "eye dominance" can be traced back to Kepler ( 20 ) determination that visual direction is set by an optical line from the viewed object to the retina. This determination was considered undisputed, as the eyes are the ultimate source of vision ( 21 ). Later theorists argued that direction is not only determined by an optical line to the retinas, but is determined by an optical line to the retina of the dominant eye ( 22, 23 ). Their view was supported by repeated empirical observations that individuals align targets with one eye and not the other, for instance, in Dolman's peephole test (e.g., 24, 25, 26 ). This eye is considered to be the dominant one.

Subsequent studies supported the concept of eye dominance, demonstrating a preference for one eye over the other. For instance, one of the eyes usually suppresses sensory input from the other in case of rivalry inputs. Next, visual acuity is sometimes better in one of the eyes and not the other and finally, there is better sensory motor coordination with one eye than with the other (See reviews by 27, 28 ). However, the concept of eye dominance has also suffered considerable criticism when repeated empirical investigations demonstrated that the interrelationships between different measures of dominance are very low (See reviews by 15, 28 ). Further, it was also demonstrated that dominance might even change with the same measure when task characteristics are different ( 29 ). Finally, a series of sophisticated experiments demonstrated that even though sighting or alignment of targets is usually done to a sighting eye, egocentric visual direction is closely associated with the "cyclopean eye" (e.g., 14, 28, 30, 31 ).

It appears, then, that the possible role of the dominant eye in vision had not been strongly established yet. Still, researchers strongly point to the hemispheric laterality that characterizes other established phenomena as handedness or footedness, as a possible source for "eyedness" or eye dominance. For instance, in a large meta-analysis Bourassa, Mcmanus ( 27 ) convincingly showed strong relationships between measures of eye dominance and measures of hand and feet dominance. These relationships may suggest that dominant eyes may be superior to nondominant eyes in certain tasks, just as dominant hands or feet are ( 27 ). This view, in turn, has gained some support from empirical findings.

For instance, Han, Seideman ( 32 ) showed that dominant eyes (i.e., the "sighting" eyes in tests like Dolman's) make more accurate vergence movements in response to different viewing conditions. In Van Leeuwen, Westen ( 33 ), individuals sometimes preferred to make short saccades to nearby objects with only their dominant eyes. Next, in Moiseeva, Slavutskaya ( 34 ), pre-saccadic processes appeared earlier in the dominant than in the nondominant eye, possibly suggesting faster sensory processing and attention disengagement for the dominant eye. Finally, Kawata and Ohtsuka ( 35 ) showed that when individuals tracked an X shaped target moving on a rail at different speeds, vergence movements were first initiated with the dominant eye and were faster with the dominant eye than with the non-dominant eye.

It seems, then, that dominant eyes may have certain qualities in some tasks and thus, although the collective evidence in support of eye dominance is currently not very strong, it is possible that dominant eyes will still be more accurate in motor tasks such as the tracking of targets.

### The question of what eye should be the input device in gaze interfaces

The question of whether it is the cyclopean or the dominant eye that fixates more accurately on targets has theoretical significance, but also practical implications for the design of gaze interfaces. Efficient human-computer interaction requires rapid and seamless capturing of on-screen targets to avoid missed commands and long selection times ( 1, 10, 11 ). Vidal, Bulling ( 36 ), developed a promising technique in this respect-‘Pursuits’ that is based on the similarity of trajectories between the eye and the object it pursues. When the correlation coefficient between a sample of the eye and object coordinates is greater than a predefined threshold, ‘Pursuits’ detects that the object is being pursued. Usability tests of Pursuits-based interaction, when users interacted with circular and linear-trajectory objects, showed high percentage of successful detections.

The most widely used technique of gaze interaction, to date, with both, stationary and moving objects, is gazebased interaction. That is, users can select and interact with objects at times when they point at them with their eyes. Therefore, testing what eye-input method is most accurate may assist in facilitating more successful gaze-based user interaction. In the current study, we compared tracking performance between the dominant, non-dominant and cyclopean eye.

## Experiment 1: Exploratory study

The purpose of the first experiment was to obtain first impression on what eye tracks a moving target more accurately before we test this question with gaze-interface tracking.

### Method

#### Participants

27 undergraduate psychology and engineering students participated in the experiment in partial fulfillment of the requirements of a course in human factors engineering. Age ranged from 21 to 31 years (Mean=26, SD=2.7). 48% of the participants were males. We tested participants for normal binocular vision using Snellen test and for binocular stability using the “Parallel infinity balance test” (PTIB) ( 37 ).

Participants' ocular dominance was tested using the Dolman's Hole in the card/Peephole test (e.g., 24, 25, 26 ). 19 of the 27 participants (70 %) were right-eyed. 24 of the 27 participants (89%) were right-handed. 6 of the 27 participants (22%) had an opposite eye-hand lateral dominance (i.e. right dominant eye with left dominant hand and vice versa). All mentioned proportions comply with the proportions reported in Bourassa, Mcmanus ( 27 ) metaanalysis.

#### Task and procedure

Participants arrived at the lab for individual sessions that lasted approximately 20 minutes. Upon arrival, they were briefed about the procedure by the experimenter that encouraged participants to ask questions throughout and after the briefing. Participants signed the informed consent form only after the experimenter confirmed that they understood the procedure. Then, the experimenter tested participants for normal binocular vision and eye-dominance. The experiment was conducted in a sound-attenuated and darkened room. Participants sat in front of the display screen and the binocular eye tracker`s desktop camera (“Eyelink 1000” see apparatus).

**Figure 1:**
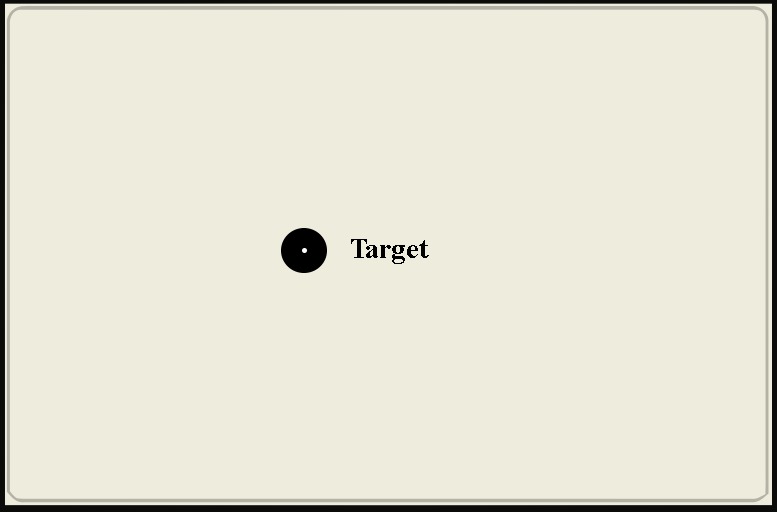
The experimental task.

Participants performed a free gaze-tracking task (see Figure 1). They were instructed to “track the moving target with their eyes”. The moving target was a red circle, 80 pixels in diameter and 1.87° from a viewing distance of 65cm. Mean percent time on target of a similar size in a previous study we conducted with joystick tracking was approximately 55% ( 38 ) and we therefore anticipated that participants in the current study would be able to track the target with their eyes. We created six tracking conditions: 3 target velocities X 2 maneuvering types. Target velocities were: 1.7°/sec, 3.1°/sec and 4.5°/sec. Maneuvering types were straight lines and curved lines. Lowest and medium velocities were also adapted from Wagner, Sahar ( 38 ) and maneuvering types were chosen to create lower (straight lines) and higher (curved lines) degrees of difficulty ( 39 ).

**Figure 2:**
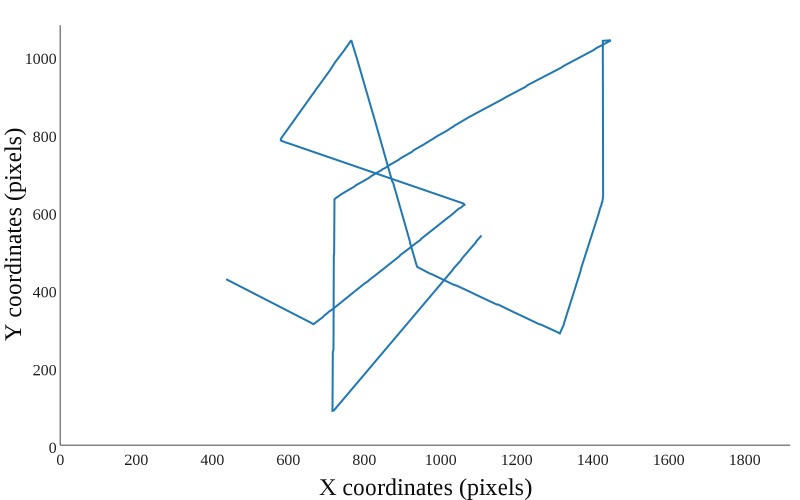
Straight lines maneuvering types.

**Figure 3:**
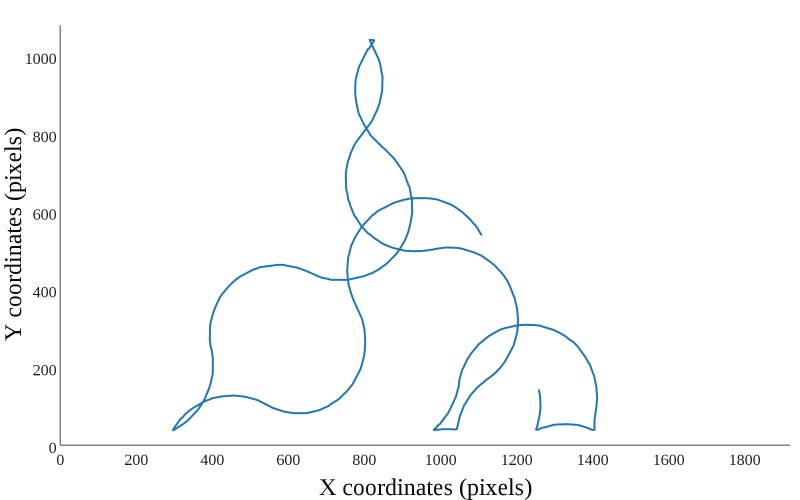
Curved lines maneuvering types.

In the straight lines maneuvering type, the target moved in a straight path, changing angles every 2-5 seconds. The experimental program randomly selected both, angle size and timing of turns. In the curved lines maneuvering type, the target moved along a curve, yet every 2-5 seconds it made a turn and started moving along a new curve. In terms of the experimental program, curves were arcs of circles with radii of 200-600 pixels and it randomly selected the radius of circles and the timing of turns. In both the straight and curved lines movement, whenever the target hit the edges of the monitor it turned to the opposite direction in a similar angle as the impact angle, relative to the perpendicular. Figure 2 and 3 show an example of curved and straight lines movements. The different maneuveringvelocity combinations allowed us to test our hypothesis across six different movement profiles as summarized in Table 1. Each profile was equivalent to a single experimental trial of 45 seconds. The experiment was composed of 2 blocks. Each block contained 6 trials of 45 seconds according to the 6 movement profiles in Table 1. The order of trials in each block was randomized. Overall, participants performed 12 trials, experiencing each tracking condition (i.e., movement profile) twice, once in each block.

**Table 1:** The 6 tracking conditions within an experimental block according to 3 velocities X 2 maneuvering types

		**Velocity**	
**Maneuver**	Slow (1.7°/sec.)	Medium (3.1°/sec.)	Fast (4.5°/sec.)
Straight Lines	Slow & Straight	Medium & Straight	Fast & Straight
Curved Lines	Slow & Curved	Medium & Curved	Fast & Curved

#### Apparatus

Data Collection and Stimulus Presentation: Binocular eye-movements were tracked with the EyeLink 1000 system (SR Research Ltd., Mississauga, Canada) with a sampling rate of 250Hz. To avoid head movements and to ensure a constant viewing distance of 65 cm, participants rested their chins on a rest with a forehead support band. We performed a calibration procedure based on a ninepoint grid at the beginning of each block using the manufacturer’s software. It was a binocular calibration, yet the mathematical models of gaze positions were fitted to each eye independent of the other as described in Stampe ( 40 ) and in accordance with previous studies with binocular measurements ( 41, 42 ). Practical calibration error was 23.24 (SD=6.37) and 22.75 (SD=7.31), in minutes of arc, for the left and right eye, respectively. Following each trial, we performed a “drift correction” procedure, where participants fixated on a calibration point for a few seconds while the system corrected any drifts it had from initial calibration.

Two interfaced computers managed the data collection and stimulus presentation in the experiment: the Eye-Link1000 host computer and the task computer. The task computer controlled stimulus presentation and managed task intervals via self-developed software (C#). Stimulus (moving target) was presented on an Alienware OptX AW2310, 23'' monitor with 1920 x1080 resolution and a 120 Hz refresh rate. The Eye-Link 1000 host computer was set as the main experimental computer, coordinating and recording all aspects of the experiment.

#### Design

Tracking performance was the dependent variable. It was quantified as the "mean absolute distance" between eye and target measured in minutes of arc (usually termed arc min). "Mean absolute distance", often referred to as "mean absolute error" is a common measure of tracking performance ( 43 ). It is calculated by aggregating eye to target distances across all samples in a trial, and dividing this aggregated sum by the number of samples in that trial, as shown in Formula 1. The higher the mean absolute distance between eye and target positions, the lower tracking performance is.

**Figure:**
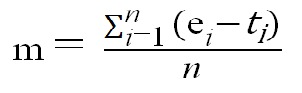


Where: m = Mean absolute distance in minutes of arc 

*n* = Number of samples for each trial 

*i* = Sample index 

e = Eye position

t = Target position

We computed tracking performance separately for the dominant, non-dominant and cyclopean eye.

The four independent variables in the experiment were: *Eye classification*: Dominant, non-dominant and cyclopean eye. *Target velocity*: 1.7°/sec, 3.1°/sec and 4.5°/sec. Maneuvering type: straight or curved lines. Experimental block: first block or second block.

This yielded a 3 X 3 X 2 X 2 within subjects design. Cyclopean eye was defined as the averaged x-y coordinates of the dominant and non-dominant eye.

#### Results

As a preliminary step to our analyses, certain data had to be excluded for being irrelevant for our study. Participants in our study were essentially engaged in a smooth pursuit task. However, the purpose of our study was not to investigate the underlying mechanisms of smooth pursuit. Rather, we aimed to compare tracking accuracy between dominant, non-dominant and cyclopean eyes to learn about expected performance in gaze control interfaces. Therefore, saccades, that for all participants, constituted attempts by the oculomotor system to recapture targets that moved outside their foveae ( 44 ), had to be regarded as noise and be filtered out. Essentially, eye-to-target distance during a saccade is irrelevant for studying gaze control, because visual information processing is largely suppressed during saccades ( 45, 46 ) and thus, very little control (if any) is possible. In this respect, our research resembles the study of eye movements in real-life reading conditions, where in many instances saccades are regarded as noise ( 47 ).

To identify saccades, we used the online SR research event detection algorithm, which is the most widely used event detection algorithm for academic research ( 47 ). The algorithm was set according to the following parameters: saccadic velocity threshold of 30°/sec, saccadic acceleration threshold of 8000°/sec, saccadic motion threshold of 0.2°. This setting is considered a conservative one and is widely used in eye-movement research ( 48 , pp. 89-94). Data exclusion procedure resulted in filtering out ~ 8.00% of the original data.

Finally, we used Linear Mixed Models (LMM) in all statistical analyses. LMM is recommended for eye tracking data that are often unbalanced due to instances where trackers fail to capture participants' eyes ( 38, 47 ).

##### Tracking Accuracy

To compare tracking accuracy between the dominant, non-dominant and cyclopean eye, we conducted a Linear Mixed Model (LMM) analysis with a random intercept on the mean distance from target. The random effect was the participants themselves and the fixed effects were eye classification (dominant, non-dominant and cyclopean), target velocity (1.7°/sec, 3.1°/sec and 4.5°/sec), maneuvering profile (straight or curved lines) and experimental block (first block or second block). We included all second-, third-, and fourth-order interactions between the fixed effects in the model.

**Figure 4:**
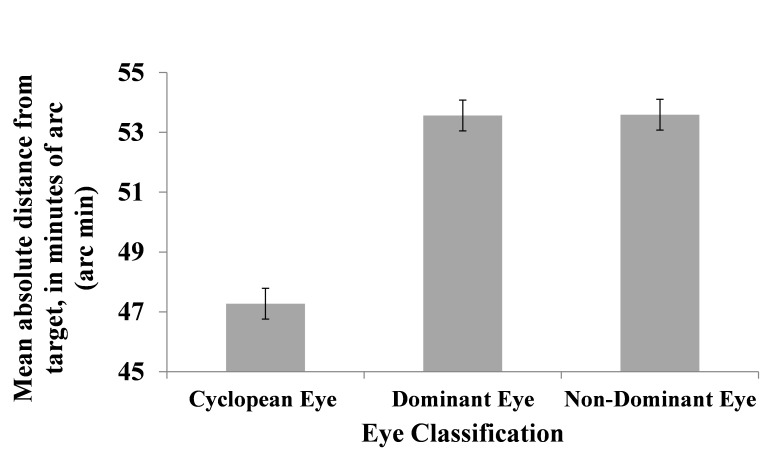
Mean absolute distance from target with cyclopean, dominant and non-dominant eyes. Error bars represent standard errors.

Our analysis of tracking accuracy showed that mean absolute distance from target was smallest with the cyclopean eye (Mean=47.27 arc min, SE=1.03 arc min). We also found that mean distance from target with dominant and non-dominant eyes was almost similar (Mean=53.56 arc min, SE=1.03 arc min; Mean=53.59 arc min, SE=1.03 arc min, respectively). Figure 4 summarizes these means and SEs. The main effect for "eye classification" was significant, F (2, 810) = 12.174, p<.001. Subsequent pairwise comparisons using Sidak correction revealed significant differences between the cyclopean and both the dominant and non-dominant eyes (p<.05). Thus, findings show that, on average, the cyclopean eye was closest to target. Finally, no significant differences in mean distance from target were found between dominant and non-dominant eyes.

Velocity also affected tracking accuracy. Mean distance from target was highest when velocity was greatest (Mean=75.64 arc min, SE=1.03 arc min), smaller for medium velocity (Mean=51.25 arc min, SE=1.03 arc min) and smallest for lowest velocity (Mean=46.66 arc min, SE=1.03 arc min). Main effect for "velocity" was significant, F (2, 810) = 22.307, p<.001. All multiple comparisons between levels of velocity, using sidak correction were also found significant (p<.05). Thus, the faster the target moved the more difficult it became tracking it. No other significant effects were found.

#### Discussion

Findings in Experiment 1 showed that cyclopean gaze positions were closest to target. We also found that velocity, but not the maneuvering profile affected tracking accuracy. Although the timing of turns and radii in the curved lines maneuvering type were random, the target still travelled within a constant radius along the curve and its path, therefore, could have been relatively predictable. Predictability, in turn, may lead to similar performance for different maneuvers (e.g., straight vs. curved lines), while shifts from one constant velocity to the next still generate changes in performance ( 49 ). Such pattern, where velocity affects performance when changes in path do not, corresponds with our findings.

Our main finding regarding the higher accuracy of cyclopean gaze positions replicates Cui and Hondzinski ( 12 ) findings and extend them to moving targets. They also correspond with the fixation disparity phenomenon where “real” eyes sometimes miss targets ( 18, 19 ). From a theoretical perspective, our findings lend further support to the cyclopean eye theory of egocentric direction. Essentially, it appears more likely that visual direction is determined according to a locus that is more often aligned with the target, than according to another locus (i.e., the dominant eye) that is less often aligned with the target. Our findings, however, did not support the hemispheric laterality approach that dominant eyes may be superior to non-dominant eyes in certain tasks ( 27 ). From a practical perspective, the higher accuracy with the cyclopean eye may suggest that performance with gaze interfaces should be better when cyclopean eye is the input device. At the same time, however, Experiment 1 was a preliminary investigation with free tracking and therefore, the implications of our findings for actual gaze control should be further investigated.

One important question pertains to the difference in percent time on target between cyclopean and single-eye control. If we were to set a perimeter that designates when users can interact with the target (e.g., a crosshair that designates that they are on target), how often would this perimeter overlap with the target with cyclopean compared to single-eye tracking? Findings from Experiment 1 showed that the average difference in accuracy between the cyclopean and real eyes was ~6 arc minutes and therefore, smaller than the calibration error we reported in the Method (23.24 and 22.75 arc min, for the left and right eye, respectively). Thus, although the mean difference in accuracy between the eyes that we computed on an extremely large sample (sampling rate was 250Hz) is robust, calibration error suggests that single measurements may sometimes be biased in favor of one eye or the other. Such bias may even increase near the edges of the monitor. One should therefore test how often the tracker indeed detects the cyclopean eye closer to the target than the other eyes, so that it can interact with the target while the other eyes cannot. The frequency of such instances can be tested if one tries to place a crosshair or cursor on target.

Second, one may indeed have a cursor or a crosshair when using gaze interface, as one usually has when she or he are operating a computer mouse or a joystick. Alonso, Causse ( 1 ) tested gaze control in ATC (air traffic control) and found that target selection accuracy has greatly improved when users received feedback on their gaze positions. At the same time, however, Jacob ( 50 ) noted that when cursor and target do not completely overlap, as a result of system errors, users may turn their attention to the cursor instead of gazing at the target. It is thus unclear how cyclopean control would compare to single-eye control if eyes sometimes pursue the cursor instead of the target. In Experiment 2 we compared cyclopean to single-eye control in gaze interface tracking.

## Experiment 2

Based on Experiment 1 results, we designed a follow up study where users tracked a target with a crosshair.

### Method

#### Participants

All participants from Experiment 1 (see Participants sub-section of Experiment 1) also participated in Experiment 2 after one to five days interval.

#### Task and Procedure

**Figure 5:**
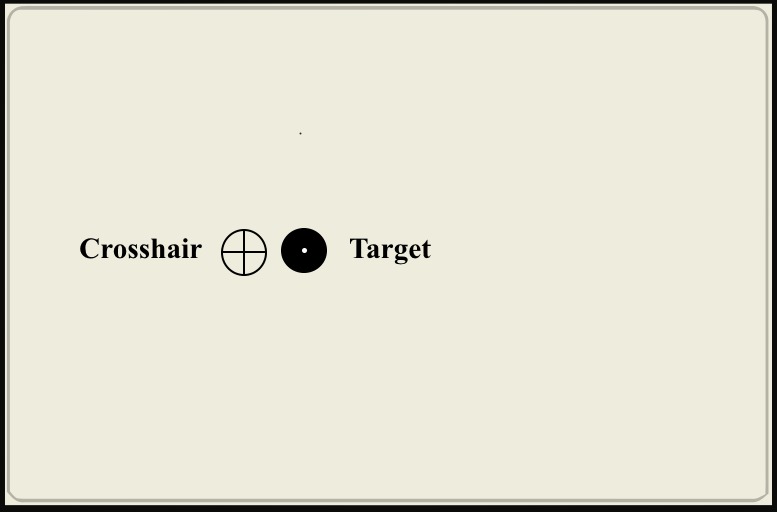
The experimental task.

Similar to in Experiment 1, participants arrived at the lab for individual sessions. Upon arrival, they were briefed about the procedure by the experimenter and were encouraged to ask questions throughout and after the briefing. Participants signed the informed consent form only after the experimenter confirmed that they understood the procedure. The task was identical to Experiment 1 in that participants had to track a moving target. However, different from Experiment 1, where we examined tracking in free gaze conditions, in Experiment 2 participants performed the tracking task with a gaze-interface. This meant that participants tracked the target with a crosshair (see Figure 5) and were instructed to “track the moving target with the crosshair”. The experimenter explained to them that they controlled the crosshair with their eyes.

**Figure 6:**
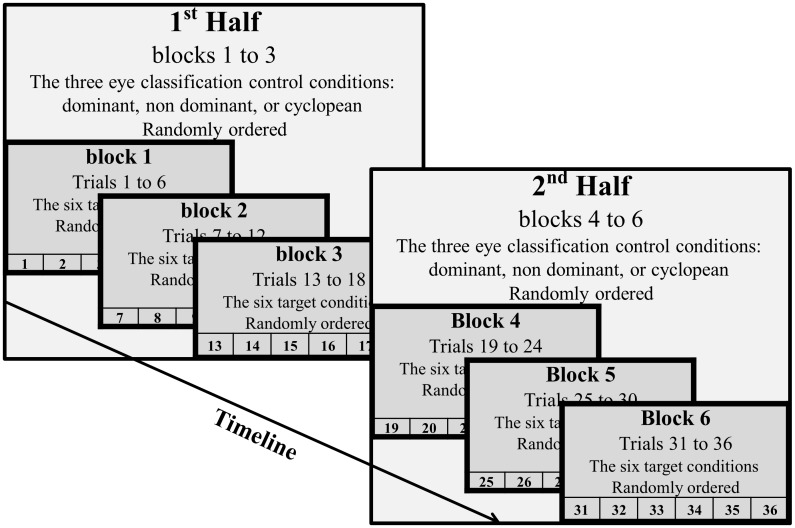
Experimental structure.

Experiment 2 was composed of six blocks as shown in Figure 6. Each block contained the six tracking conditions as in Experiment 1 (3 velocities X 2 maneuvers). In each block, the crosshair was controlled by either one of the eyes, according to the three eye classification categories: dominant, non-dominant, and cyclopean eye. Hence, participants experienced each of the three eye-crosshair coupling conditions twice. We randomized the order of eyecrosshair coupling across blocks. However, complete randomization could have resulted in sequences where the same eye controls the crosshair in the last two or the first two blocks. Such instances could have led to training effects, and thus, to a possible confounding in our results. In other words, such instances could have caused enhanced training prior to some eye classification conditions, while generating no training prior to other eye classification conditions. Therefore, we chose to perform semi-randomization.

Essentially, we did not randomize all 6 blocks as a group, but rather, decided to define the first three blocks and the second three blocks as two halves, as depicted in Figure 6, each of them with all three control options (dominant, non-dominant, and cyclopean). Then, we randomized the first three blocks and the second three blocks separately. This way, there were no sequences where the same eye controlled the crosshair in the last two or first two blocks. Following the first half of the experiment, participants received a five-minute break.

Although participants knew they controlled the crosshair with their eyes, they were not informed which of the eyes controlled the crosshair in each block. This was because we were concerned that such information may disrupt participants' natural interaction with the interface. Essentially, users in real-life settings are not expected to think about how they move their eyes to interact with gaze interfaces ( 50 ). After completing six blocks, the experiment ended. The experimenter briefed participants about the main research questions and thanked them for their participation. The entire procedure lasted approximately 45 minutes.

#### Apparatus

The apparatus in Experiment 2 was identical to in Experiment 1 except for activating an additional software function. In each block, the experimental software coupled the crosshair to one of the three eyes (dominant, non-dominant, or cyclopean). This function enabled us to compare gaze interface tracking performance between the three eyes. Calibration and drift correction procedures were also identical to in Experiment 1. Practical calibration error was 24.11 (SD=7.03) and 23.77 (SD=7.56) arc min, for the left and right eye, respectively.

#### Design

 The dependent variable was the percent of time crosshair and target overlapped (termed “percent on target”). Percent on target is often used as a measure for tracking accuracy when using a crosshair ( 43, 51, 52 ). To estimate percent time on target, crosshair was tagged “on” for every data sample crosshair and target overlapped (partly or fully) and “off” when crosshair and target did not overlap, where “on”=1 and “off”=0 ( 52 ). Then, sample values were aggregated and divided by the number of samples, as demonstrated in formula 2. This measure allowed us to estimate the percent of time during each trial that participants succeeded in “capturing the target”.

**Figure:**
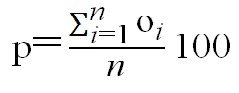


Where: p = Percent Time on Target

*n* = Number of samples in trial

*i* = Index of sample

o = "On Target" (binary variable)

o_i_=1 if target and crosshair overlap (partly or fully) 

o_i_=0 if target and crosshair do not overlap 

The four independent variables in the experiment were: Eye classification: Dominant, cyclopean or non-dominant eye. Target velocity: 1.7°/sec, 3.1°/sec or 4.5°/sec. Maneuvering type: straight or curved lines. Experiment half: first half or second half. This yielded a 3 X 3 X 2 X 2 within subjects design.

### Results

Data exclusion was similar to Experiment 1 and resulted in similar proportion of excluded data (~8%).

#### Tracking Accuracy

We conducted a Linear Mixed Model (LMM) analysis with a random intercept on "percent time on target". The random effect was the participants themselves and the fixed effects were eye classification (dominant, non-dominant and cyclopean), target velocity (1.7°/sec, 3.1°/sec or 4.5°/sec), target maneuver (straight or curved lines) and half of the experiment (first half or second half). We included all second-, third-, and fourth-order interactions between the fixed effects in the model.

Greatest percent on target was achieved when crosshair was controlled by the cyclopean eye (Mean=62.13, SE=1.42) compared to when crosshair was controlled by either the dominant (Mean=55.95, SE=1.38), or the nondominant eye (Mean=54.04, SE=1.36). The main effect for "eye-classification" was significant, F (2,797) = 9.10, p<.001. Subsequent pairwise comparisons using Sidak correction revealed significant differences between the cyclopean and both the dominant and non-dominant eye (p<.01). We found no significant differences in percent time on target in the pairwise comparisons between the dominant and non-dominant eye.

**Figure 7:**
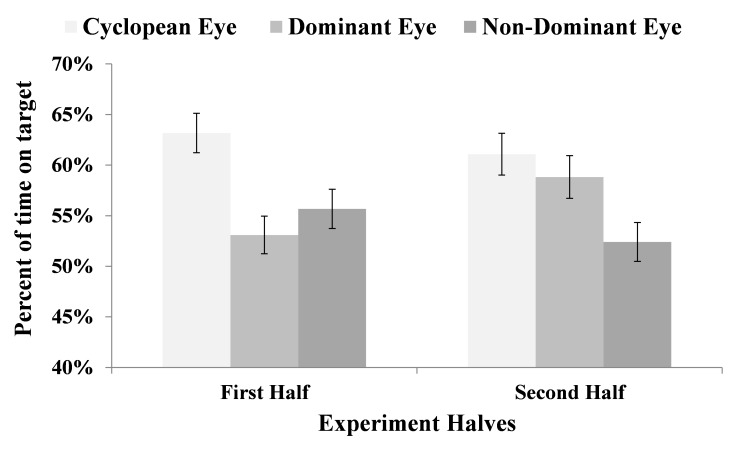
Tracking-performance with cyclopean, dominant, and non-dominant eye-control in the first and second halves of the experiment. Error bars represent standard errors.

We found a significant interaction Experiment half X Eye classification, F (2,797) = 3.12, P<.05. Figure 7 demonstrates that while differences in mean percent time on target between cyclopean and the two other eyes were quite large in the first half of the experiment, mean percent time on target became more similar between cyclopean and dominant eye in the second half of the experiment (Mean=61.08, SE=2.06 and Mean=58.82, SE=2.11, respectively). Pairwise comparisons using Sidak correction revealed no significant difference between cyclopean and dominant eye in percent time on target in the second half of the experiment. It was only the difference between cyclopean and non-dominant eye that was statistically significant (Mean=61.08, SE=2.06 and Mean=52.41, SE=1.92, respectively), (p<.01).

To test whether dominant eye tracking had indeed significantly improved between the first half (Mean=53.09, SE=1.8) and second half of the experiment (Mean=58.82, SE=2.11), we ran a Linear Mixed Model (LMM) analysis, quite similar to the first one, yet, this time, on the dominant eye alone. In other words, eye classification was not an independent variable in this model because it had only one level (i.e., only the dominant eye). We found that improvement in dominant-eye tracking was indeed significant, F (1, 269) = 4.96, p< .05.

Last, using again the full model we described at the beginning of the Results section, we also found that percent on target was highest when target traveled at 1.7°/sec (Mean=60.36, SE=1.39), less when target traveled at 3.1°/sec (Mean=58.23, SE=1.38), and smallest when target traveled at 4.5°/sec (Mean=53.52, SE=1.38). The main effect for "Velocity" was significant, F (2,797) = 6.33, P<.01. Yet, subsequent pairwise comparisons using Sidak correction revealed a significant difference only between the greatest and smallest velocities (4.5°/sec vs. 1.7°/sec), (p<.01). No other significant main effects or interactions were found.

### Discussion

The main finding in Experiment 2 replicated the main finding in Experiment 1, namely, that tracking accuracy was best with the cyclopean eye. Thus, we expect cyclopean tracking to be more accurate than single-eye tracking also in cases where eyes control a crosshair. In contrast to Experiment 1, however, findings in Experiment 2 did indicate that dominant eyes might have unique qualities in motor tasks. In the General Discussion, we present a wider theoretical view of our findings and discuss the possible limitations of this study.

## General Discussion

We tested which of the eyes would lead to greatest accuracy when tracking a moving target: the dominant eye, the non-dominant eye, or the metaphorical "cyclopean eye" that we embodied its estimated projection by averaging the x-y coordinates of the two real eyes. Findings from both Experiment 1 and Experiment 2 showed that cyclopean-eye tracking would be the most accurate as the mean cyclopean distance from target was the smallest in Experiment 1 and mean percent time on target was highest with the cyclopean eye in Experiment 2. At the same time, however, a significant interaction between eye classification and the half of the experiment in Experiment 2 suggested that supremacy of the cyclopean eye was limited to the first half of the experiment. These findings have both theoretical and practical implications.

From a theoretical view, our findings replicate Cui and Hondzinski ( 12 ) findings that the average gaze positions of the two eyes is closer to targets than the single gaze positions of either eye alone. These findings resonate with the cyclopean eye theory of egocentric direction (e.g., 14, 15, 16, 17 ). They also show that average gaze positions (or “cyclopean positions”) are not only closer to stationary targets as in Cui and Hondzinski ( 12 ), but also to moving targets.

In addition, our findings provide indication for eye dominance, in accordance with the hemispheric laterality approach that dominant eyes may be superior to non-dominant eyes in certain tasks, just as dominant hands or feet are ( 27, 53 ). As we mentioned in the Introduction, the idea of hemispheric laterality with respect to ocular dominance has suffered great criticism (e.g., 15, 29 15, 29), yet a number of empirical reports did show indications for it ( 32-35 ). This also seems to be the case in the current empirical report.

Tracking accuracy with the dominant eye in Experiment 2 improved with time and became more similar to tracking accuracy with the cyclopean eye. No such improvement was found in Experiment 1 that included only two blocks and no such improvement was found with the non-dominant eye in neither experiments. Thus, training improved performance, yet only with the dominant eye. It appears, therefore, that evidence of asymmetric motor performance between the dominant and non-dominant eye is accumulating and we believe that further empirical investigations of this phenomenon are highly necessary.

The practical implications of our study relate to the design of gaze interfaces. We showed in two experiments that cyclopean tracking is more accurate than single-eye tracking and therefore, designers of gaze-interfaces may want to consider cyclopean control. Tracking accuracy will of course depend on task characteristics, as for example, the size of targets. The mean difference in percent time on target between cyclopean and dominant-eye tracking in the task we designed for Experiment 2 was ~6% and would probably increase with smaller targets and decrease with larger targets. Our main interest in this study was in the question of whether the effectiveness of gaze interaction may depend on what eye one uses as the input device. We therefore used a relatively small target and did not explore the relative effects of different target sizes and other task characteristics that may possibly affect tracking accuracy. Designers of gaze-interfaces should decide what eye to use as the input device according to the characteristics of the task and the rewards and punishments for different outcomes. For instance, would 6% difference (or less/more) in percent time on target be enough to justify cyclopean control for reducing missed commands and selection times in a video game? What about reducing missed commands and selection times in ATC or in combat piloting? Our study does not provide answers to these questions. It shows that what eye to use as input should be a design consideration in gaze interfaces.

We focused in this study on tracking, where gaze control holds great promise in replacing the less natural tracking with joystick or with a mouse, while at the same time it has been reported to call for methods to improve accuracy (e.g., 10, 11 ). In addition, our task did not require participants to select targets, for instance by pressing a bar ( 7 ), or by waiting a predefined dwell time before selection ( 3, 54 ). We demonstrated that crosshair and target overlapped for a greater percentage of time with cyclopean compared to single-eye control and therefore, that selection of targets should reasonably be faster in such conditions. Cyclopean fixations are also expected to be more accurate when focusing on stationary targets ( 12 ) and not only when targets are moving. In future studies we intend to compare cyclopean and single-eye control when users select targets and when targets are stationary (e.g., on-screen icons). Future studies should also test the relative accuracy of the eyes in free interaction, when users move their heads. Tracking error in such cases can sometimes exceed 1.5^0^ ( 55 ) and one should therefore test whether the distribution of errors does not bias the position for one eye more strongly than for the other.

In addition, we invited participants to single experimental sessions and we therefore could not assess whether they retained any skill in eye tracking they may have acquired during the experiment. Being able to retain such skill may imply that expert users of gaze control interfaces will be equally accurate when capturing targets with their dominant as with their cyclopean eye. Future empirical investigations should look at the longer-term effects of training on target-capturing accuracy with gaze control interfaces.

Last, our estimated projection of the cyclopean eye was based on an unweighted average of the x-y coordinates of the dominant and non-dominant eye. However, a weighted average with greater weight for the dominant eye would have inevitably driven the crosshair closer to target in the second part of the experiment where dominant control improved. Gaze-interface interaction may therefore benefit from the development of more sophisticated eye-crosshair coupling algorithms with an alternating weighting system according to real time data about tracking accuracy.

## Conclusions

In two experiments, we demonstrated that tracking accuracy was better with the cyclopean eye than with the dominant and non-dominant eye. We also showed similar tracking accuracy with the cyclopean and dominant eye in the second half of Experiment 2. Our findings correspond with the cyclopean eye theory of egocentric direction and provide indication for eye dominance, in accordance with the hemispheric laterality approach. From a practical viewpoint, we showed that what eye to use as input should be a design consideration in gaze interfaces.

## Acknowledgements

We wish to thank Lior Lahav for developing and implementing all programming aspects of this project.

The authors declare that there is no conflict of interest regarding the publication of this paper.
